# Determination of glyphosate and AMPA in freshwater and soil from agroecosystems by 9-fluorenylmethoxycarbonyl chloride derivatization and liquid chromatography - fluorescence detection and tandem mass spectrometry

**DOI:** 10.1016/j.mex.2022.101730

**Published:** 2022-05-13

**Authors:** Beatriz Alonso, Luciana Griffero, Heinkel Bentos Pereira, Lucía Pareja, Andrés Pérez Parada

**Affiliations:** a Departamento de Desarrollo Tecnológico, Centro Universitario Regional del Este, Universidad de la República, Uruguay; b Departamento de Química del Litoral, Centro Universitario Regional Litoral Norte, Universidad de la República, Uruguay

**Keywords:** glyphosate, AMPA, environmental fate of pesticides, fluorescence detection (FLD), tandem mass spectrometry (MS/MS), liquid chromatography (LC), FMOC-Cl

## Abstract

The herbicide glyphosate (GLY) and its metabolite aminophosphonic acid (AMPA) are troublesome compounds for analysis in the environment. Here we report a reliable technique for GLY and AMPA determination in freshwater and soils by means of derivatization with 9-fluorenylmethoxycarbonyl chloride (FMOC-Cl) and further liquid chromatography with fluorescence detection (FLD) and tandem mass spectrometry (MS/MS) analysis. Selected experiments were carried out to evaluate selectivity, sensitivity, repeatability, linearity and quantification performance in both matrices.

## Highlights

• simplified and high throughput sample preparation for freshwater and soil
• robust and optimized instrumental conditions for LC-FLD and LC-MS/MS analysis
• application to > 100 real samples

## Graphical abstract

**Figure:**
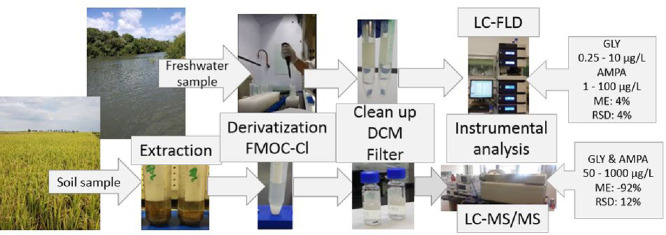

Specification table

**Table:** 

Subject Area;	*Environmental sciences*
More specific subject area;	*Pesticide residues in environment*
Method name;	*Glyphosate and AMPA in freshwater and soil by FMOC derivatization and fluorescence and tandem mass spectrometry detection*
Name and reference of original method;	*ISO 21458:2008 (glyphosate and AMPA in water)*
Resource availability;	*Not applicable*

## Method details

### Chemicals and reagents

Glyphosate (GLY) (99.4%), aminophosphonic acid (AMPA) (99%) and glyphosate-FMOC (GLY-FMOC std) (98%) reference standards and the derivatizing reagent FMOC-Cl (9-fluorenylmethoxycarbonyl chloride, 99%) were purchased from HPC Standards GmbH (Cunnersdorf, Germany). HPLC-grade dichloromethane (DCM) acetonitrile (MeCN) and methanol (MeOH) were supplied by Merk (Darmstadt, Germany). Ultrapure water was obtained from a Smart2Pure 3 UV from Thermo Scientific (Massachusetts, EEUU). Stock solutions of GLY and AMPA were prepared with ultrapure water from the standard substances at 2000 mg L^−1^. Working solutions were prepared by appropriate dilution of the stock solutions in ultrapure water. The derivatizing solution was prepared in MeCN at 6 g L^−1^ (23 mM) of FMOC-Cl. The working and derivatizing solutions were stored in darkness at 4 +/-2°C. Sodium borate decahydrate (Na_2_B_4_O_7_ 10H_2_O), potassium dihydrogen phosphate (KH_2_PO_4_), ammonium acetate (NH₄CH₃CO₂) and ammonium hydroxide (NH_4_OH) of analytical grade were supplied by Carlo Erba (Cornaredo, Italy). Solutions of these reagents were prepared separately in ultrapure water at the following concentration levels: 100, 10, 1.0, 0.50 mg L^−1^.

### Sampling, sample storage and blank samples

Blank samples of freshwater were obtained from a productive field located in a natural pasture livestock area (34°15′29"S 54°57′09.9"W). Soil blank samples were taken from an agricultural field (33°6′23"S, 54°10′24" W) where GLY applications have never been made. In addition, the absence of GLY and AMPA in both matrices was experimentally verified by both LC-FLD and LC-MS/MS. Full characterization of Eastern Uruguay soil matrix is described in previous works [1,2].

A representative portion sample of approximately 500 g soil sample is taken. The sample is reduced by grinding and quartering to obtain a homogeneous test sample of approximately 40 g which is freeze-dried and stored in polyethylene bags at -18°C until analysis. A representative test sample of 40 mL of water is obtained in 50 mL polypropylene centrifuge tubes and frozen at -18°C until analysis. All subsamples should be kept in darkness until analysis. No evidence of degradation was found when re-analyzing samples after 3 months of frozen storage.

### Sample treatment for freshwater samples

1) Transfer a 3.0 mL aliquot of freshwater into a 50 mL centrifuge tube and sonicate in an ultrasonic bath for 3 min.
2) Add 0.5 mL of Na_2_B_4_O_7_ 25 mM solution; 0.5 mL of FMOC-Cl 6.0 g L^−1^ (0.023 M) and 0.5 mL of MeCN. Shake and vortex for 30 s.
3) Let it react for 1 hour at room temperature (22 +/- 3 °C)
4) Add 4.5 mL of DCM. Shake and vortex for 30 s.
5) Filter 1.0 mL of supernatant through a PVDF 0.45 µm filter and collect into a 2.0 mL screw-cap vial for LC-FLD and LC-MS/MS analysis

### Sample treatment for soil samples

1) Weight 5.0 g of freeze-dried soil sample into a 50 mL centrifuge tube, add 10 mL of KH_2_PO_4_ 0.1 M solution and sonicate in an ultrasonic bath for 30 min.
2) Centrifugate at 4000 rpm for 5 minutes, filter 2.0 mL of supernatant through a PVDF 0.45 μm filter and add 2.0 ml of Na_2_B_4_O_7_ 0.1M.
3) Transfer a 3.5 mL aliquot into a 50 mL centrifuge tube and add 0.5 mL of FMOC-Cl 6 g L^−1^ and 0.5 mL of MeCN. Shake and vortex for 30 s.
4) Let it react for 1 hour at room temperature (22 +/- 3 °C)
5) Add 4.5 mL of DCM. Shake and vortex for 30 s.
6) Filter 1.0 mL of supernatant through a PVDF 0.45 µm filter and collect into 2.0 mL screw-cap vials for LC-MS/MS analysis

A general analytical workflow for both matrixes is found at Fig. S1.

### Liquid chromatography-fluorescence detection

A Thermo Scientific Ultimate 3000 LC coupled to a fluorescence detector Thermo Scientific FLD 3400RS was used for LC-FLD analysis of GLY and AMPA in freshwater samples. A Thermo Scientific Hypersil Gold C18 (250 mm x 4.6 mm id. 5 µm) column was used. The column oven temperature was set at 30°C. The mobile phase consisted of (A) 5 mM ammonium acetate buffer (pH=9.5), pH is adjusted with a diluted solution of NH_4_OH, and (B) LC grade MeCN. The separation was performed at 1mL min^−1^ with the following elution program: starts at 5% B, gradually changing until 19% B at 6 minutes and stable for 4 minutes, then to 95% in 2 minutes and keep stable for 7 minutes. The program ends by decreasing the acetonitrile (B) to 5% for column stabilization. The injection volume was 10 µL. For syringe cleaning, we used a 1mL washing volume of MeOH after each injection. The detector was operated at fixed wavelengths (λ excitation: 270 nm, λ emission: 315 nm), the FLD acquisition was programmed from 5 to 10 minutes with a sensibility factor of 2 (for GLY) and between 10 and 13 minutes a sensibility factor of 3 (for AMPA). Chromeleon v.7.2.9 software from Thermo Scientific was used for instrument control and data processing.

### Liquid chromatography-tandem mass spectrometry

LC-MS/MS analysis was performed with an Agilent 1200 LC (Agilent Technologies, Palo Alto, USA) system coupled to an AB Sciex 4000 QTRAP (Concord, Canada) quadrupole linear ion trap tandem mass spectrometer operated in scheduled MS/MS mode. The system was equipped with an electrospray (ESI) source Turbo V operated on negative ionization mode. A ZORBAX Eclipse XBD-C18 (150 mm x 4.6 mm id. 5µm) column from Agilent Technologies was used. The separation was performed at 20°C using the same mobile phase and gradient from LC-FLD analysis with a constant flow rate of 0.6 mL min^−1^. The injection volume was 5 µL. Tandem MS detection was performed using the multiple reaction monitoring (MRM) mode with negative ESI- mode. The optimal MRM conditions were optimized using direct infusion in the ESI-. Source temperature was 500°C, ionization voltage was 5000V, curtain gas was nitrogen at 20 psi and the nebulizer gas was nitrogen at 50 psi. Scheduled MRM was used with a 90 s detection window covering the expected retention time (Rt (min)) of both analytes. Analyst v 1.7.1 was used for instrument control and data processing. Table 1 describes the spectrometric conditions.

**Table 1:** LC-MS/MS conditions for both compounds. Rt: retention time; DP: declustering potential; CE: collision energy.

Analyte	Rt (min)	Precursor ion (m/z)	Product ion (m/z)	DP (V)	CE (V)
GLY-FMOC	6.5	390	168	-35	-17
	6.5	390	150	-35	-36
AMPA-FMOC	10.5	332	110	-30	-11
	10.5	332	136	-30	-23

### Optimization of instrumental conditions

We tested LC-FLD and LC-MS/MS performance. LC-FLD is a highly disseminated analytical instrumentation whereas LC-MS/MS is a standard and robust technique in most residue laboratories.

**Fig. 1:**
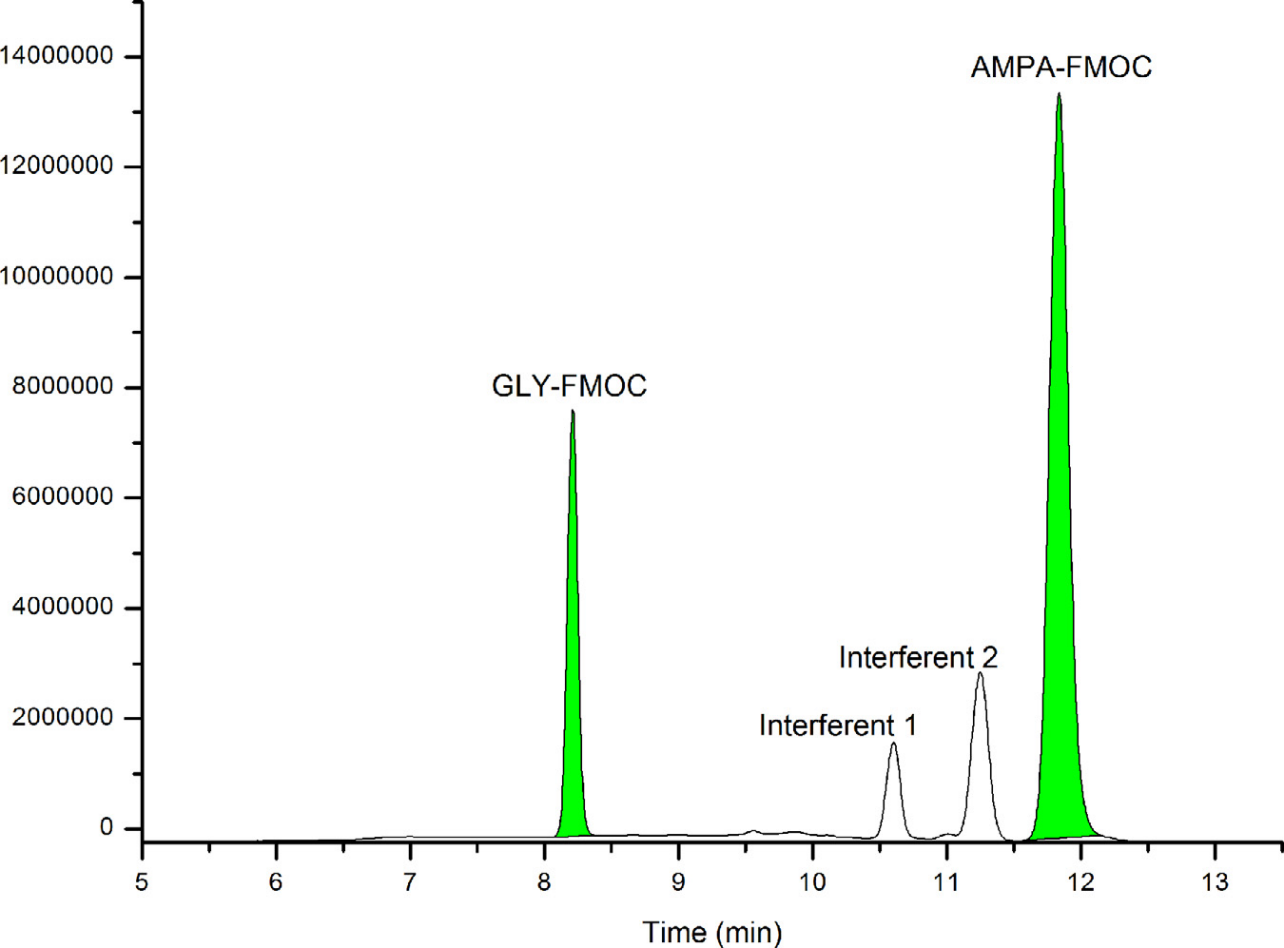
LC-FLD chromatogram of a freshwater sample at 5 µgL^-1^ GLY (Rt=8.3 min) and AMPA (Rt=11.8)

Due to the amphoteric characteristics of GLY and AMPA, we evaluated different mobile phase conditions. In the bibliography, the use of gradients in an acidic medium with the addition of formic acid (FA) [3] or a basic medium such as ammonium acetate solutions can be found [4], [5], [6]. We selected mobile phase conditions proposed by Ramirez et al. [5], in which pH = 9.5 allows GLY-FMOC and AMPA-FMOC in their anionic form, ensuring an early elution of the compounds of interest and increasing the Rt (min) of neutral interferences and FMOC by-products. This mobile phase also obtained a good chromatographic resolution (Rs) with respect to the closest interferences of the signal peaks of GLY-FMOC (Rs=13.97) and AMPA-FMOC (Rs=2.33), as shown in Fig. 1.

Retention time is pH sensitive. A soft variation of the pH of the mobile phase and Rt (min) shift was found, probably due to the volatilization of NH_3_ during prolonged analytical sequences (see Fig. S2). Therefore, the accuracy of pH adjustment of the mobile phase and its daily preparation is critical in reducing the variability in the Rt (min) of every analytical batch.

**Fig. 2:**
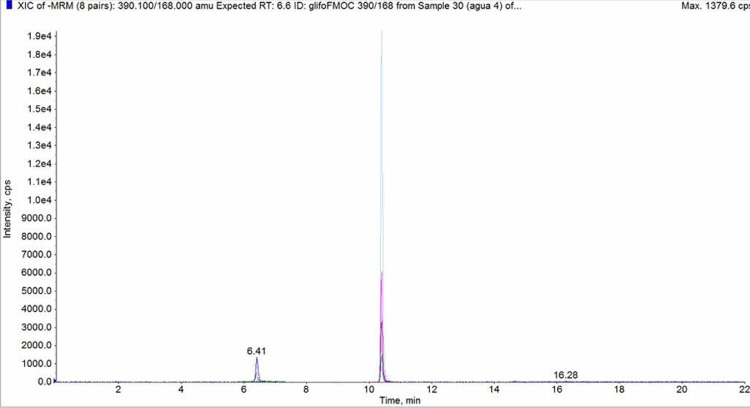
LC-MS/MS chromatogram of a freshwater sample fortified at 5 μgL^-1^ GLY (Rt=6.4) and AMPA (Rt=10.2)

Both GLY-FMOC and AMPA-FMOC are prone to carry-over effects at the injection module. This problem is minimized by using LC grade MeOH as cleaning solvent of the injection system. We also increased the flushing volume used for cleaning between injections to 1 mL after each injection. After the elution of analytes, the mobile phase gradient has a final stage in which the composition of organic solvent is maximized to wash the column and tubing of the derivatization by-products. The used FLD allows the use of different acquisition channels with variable wavelengths and sensitivity factors of the photomultiplier. These factors were adjusted for each analyte, being necessary to use a higher sensitivity factor for AMPA-FMOC detection compared to GLY-FMOC detection. Then, a good detectability was obtained for both compounds. In the case of soil, FLD detection enables GLY-FMOC determination solely. Interferences of soil-matrix reaction by-products with the same Rt (min) as AMPA-FMOC were present. The chromatographic conditions for LC-MS/MS were identical to those selected for LC-FLD (See LC-MS/MS chromatogram in Fig. 2). In this case, due to the higher selectivity of MS/MS, the long-lasting washing stage of the LC column was shortened to reduce the running time. LC-MS/MS acquisition conditions for both compounds are shown in Table 1 for the ESI negative ionization mode. These MRM conditions were optimized using GLY-FMOC y AMPA-FMOC prepared separately at the laboratory with a concentration of 1 mg L^−1^.

### Optimization of sample preparation

The use of glassware is not recommended since documented losses by adsorption [7]. We used plastic disposable materials whenever possible and glass vials after derivatization.

Different methods for GLY and AMPA analysis using FLD detection have been reported [5,7,8]. These are generally based on fundamentals of two official methods for GLY and AMPA determination in water: (i) ISO 21458:2008 which proposes derivatization in basic medium prior to chromatographic separation, detecting GLY-FMOC and AMPA-FMOC derivatives [7] and (ii) US EPA Method 547 proposed in which derivatization is performed post-chromatographic separation followed by FLD detection [9]. In this work, optimization was inspired by ISO 21458:2008 scheme. Table 2 shows different reported methods in water and soil by means of FMOC derivatization. As seen, different reports use varied customizations in chemicals and experimental conditions of analysis but also instrumental techniques including chromatographic conditions such as chromatographic columns, mobile phases, pH of mobile phases ranging from acidic to basic media but also polarities of ESI.

**Table 2:** Comparison of analytical techniques for the detection of GLY and AMPA in soil and water by FMOC-Cl derivatization. DV: derivatization; FA: formic acid; NH_4_Ac: ammonium acetate*Method validation*

Matrix	Analyte	Sample preparation	Instrumental system	Chromatographic column and Mobile phase	Quantitation approach	LOQ	Occurrence in real samples	Reference
Soil	GLY	5 g sample + KH_2_PO_4_ + Na_2_B_4_O_7_ 40 mM + FMOC-Cl (overnight DV)	LC-MS/MS positive ESI	C18 (50 mm x 2.1mm i.d. 1.7µm) gradient of A) MeOH B) NH_4_Ac 5mM	ILIS 1,2-^13^C ^15^N (GLY)	10 µg kg^−1^	35 - 1502 µg kg^−1^	[4]
	AMPA					10 µg kg^−1^	299 - 2296 µg kg^−1^	
Fresh water	GLY	2 mL sample + KH_2_PO_4_ + Na_2_B_4_O_7_ 40 mM + FMOC-Cl (overnight DV)				0.5 µg L^−1^	0.5 - 7.6 µg L^−1^	
	AMPA					0.5 µg L^−1^	0.5 - 2.6 µg L^−1^	
Groundwater	GLY AMPA	3 mL sample + HCl + KOH + Na_2_B_4_O_7_ 40 mM + FMOC-Cl + MeCN (2hs DV) + DCM cleanup	LC-MS/MS positive ESI	C18 (100 mm x 2.1 mm i.d. 1.8µm) gradient of A)H_2_0:MeCN(98:2)+0.1%FA B)MeCN+0.1%HCO_2_H	ILIS 1,2-^13^C ^15^N (GLY) ^13^C ^15^N (AMPA)	0.6 µg L^−1^	0.6 - 11.3 µg L^−1^	[3]
						0.2 µg L^−1^	0.2 - 6.5 µg L^−1^	
Freshwater, tap water and groundwater	GLY and AMPA	3 mL sample + HCl + KOH + Na_2_B_4_O_7_ 50mM + DEE + MeCN + FMOC-Cl (1h DV) + H_3_PO_3_ (stop reaction). DEE cleanup	LC-FLD	C18 (250 mm x 3 mm i.d. 5µm) gradient of A) KH_2_PO_4_ 2mM (pH=7) B) MeCN	*EC*	Not reported	Not reported	[7]
Soil	GLY	5g sample + KH_2_PO_4_ 0.1M + Na_2_B_4_O_7_ 0.1M + FMOC-Cl (overnight DV). DCM cleanup	LC-MS/MS positive ESI	C18 (50 mm x 2.1mm i.d. 1.7µm) gradient of A) MeOH B) NH_4_Ac 5mM	ILIS 1,2-^13^C ^15^N (GLY)	0.9 µg kg^−1^	0.9 - 1.3 µg kg^−1^	[10]
	AMPA					0.9 µg kg^−1^		
Rainwater	GLY	2mL sample + KH_2_PO_4_ 0.1M + Na_2_B_4_O_7_ 0.1 mM + FMOC-Cl (overnight DV). DCM cleanup				0.75 µg L^−1^	0.75 - 2.5 µg L^−1^	
	AMPA					0.75 µg L^−1^	0.75 - 7.1 µg L^−1^	
Water	GLY	20 ml sample + freeze drying + Na_2_B_4_O_7_ 25 mM + EDTA+ FMOC-Cl (3h DV). Without cleanup	LC-FLD-MS/MS	C18 (150 mm x 4.6 mm i.d. 5µm) gradient of A) MeCN B) 5mM NH_4_Ac	*EC*	0.058 µg L^−1^	0.44 - 59.9 µg L^−1^	[5]
	AMPA					0.108 µg L^−1^	1.15 - 9.09 µg L^−1^	
Fresh water	GLY	1 mL sample + Na_2_B_4_O_7_ 400 mM + FMOC-Cl +MeCN (overnight DV)	LC-MS	C18 (75 mm x 4.6 mm i.d. 3µm), gradient of A) MeOH B) NH_4_Ac 5Mm	ILIS 1,2-^13^C ^15^N (GLY)	1 µg L^−1^	17.5 - 125 µg L^−1^	[6]
	AMPA					1 µg L^−1^	1 - 4.8 µg L^−1^	
Sub surface soil	GLY	2 g sample +KOH 0.6 M + HCl + Na_2_B_4_O_7_ 5% + FMOC-Cl + MeCN (30 min) + FA conc.	LC-MS/MS negative ESI	C18 (150 mm x 2.1mm i.d. 3.5µm) gradient of A) NH_4_Ac 5Mm (pH=9) B) MeOH:H_2_O (9:1)	ILIS 1,2-^13^C ^15^N (GLY) ^13^C ^15^N (AMPA)	50 µg kg^−1^	200 - 2129 µg kg^−1^	[12]
	AMPA					50 µg kg^−1^	110 - 1270 µg kg^−1^	
Tap water	GLY	1 mL sample + Na_2_B_4_O_7_ 5% + FMOC-Cl + MeCN (30 min) + FA				10 µg L^−1^	170 - 2900 µg L^−1^	
	AMPA					10 µg L^−1^	10 - 80 µg L^−1^	
Fresh water	GLY	5 mL sample + Na_2_B_4_O_7_ 0.1M + FMOC-Cl (overnight DV) + online SPE	LC-MS/MS negative ESI	C18 (150 mm x 2.0 mm i.d. 5µm) gradient of A) (NH_4_)_2_CO_3_ B) MeOH	ILIS 1,2-^13^C ^15^N (GLY) ^13^C ^15^N (AMPA)	0.005 µg L^−1^	0.005 - 2.5 µg L^−1^	[13]
	AMPA					0.005 µg L^−1^	0.005 - 2.6 µg L^−1^	
Soil	GLY	5 g sample +KH_2_PO_4_ 0.1 M + Na_2_B_4_O_7_ 0.1M + FMOC-Cl +MeCN (1h DV).DCM cleanup	LC-MS/MS negative ESI	C18 (150 mm x 4.6 mm i.d. 5µm), gradient of A) MeCN B) NH_4_Ac 1mM (pH=9.5)	EC	50 µg kg^−1^	50 - 825 µg kg^−1^	This work
	AMPA					50 µg kg^−1^	238 - 1182 µg kg^−1^	
Fresh water	GLY	3mL sample + Na_2_B_4_O_7_ 25 mM + FMOC-Cl +MeCN(1h DV)-DCM cleanup	LC-FLD	C18 (250 mm x 4.6 mm, i.d. 5µm) gradient of A) MeCN B) NH_4_Ac 1mM (pH=9.5)		0.25 µg L^−1^	0.25 - 14.6 µg L^−1^	
	AMPA					1 µg L^−1^	1 - 36.6 µg L^−1^	
	GLY		LC-MS/MS negative ESI	C18 (150 mm x 4.6 mm, i.d. 5µm), gradient of A) MeCN B) NH_4_Ac 1mM (pH=9.5)	EC	1 µg L^−1^	-	
	AMPA					1 µg L^−1^		

Here, we combined sample preparation steps proposed by Demonte et al., 2018 for freshwater samples [3], the sample preparation proposed by Lupi et al., 2019 for soil samples [10] and combined LC-FLD and LC-MS/MSM analysis proposed by Ramirez et al., 2014 [5]. Demote et al. 2018 proposed a methodology to determine GLY and AMPA in groundwater. In that case, a preconditioning step by strong acidification of sample with HCl is mandatory to reduce the interaction of multivalent cations with the amphoteric behavior of GLY and AMPA [3]. In our study, we tested superficial freshwater from rivers and finally we have avoided this tedious step. Furthermore, this simplified strategy was tested in different freshwater sources without evidence of matrix disturbance if avoiding acidification pretreatment. Other reports present some important differences, mainly in the time and temperature required for the derivatization reaction with FMOC-Cl. Some authors have performed the derivatization with FMOC-Cl from 30 min [6] to overnight reaction [10]. We evaluated optimal conditions for reaction at 3 different temperatures (22°C, 30°C and 45°C) and reaction times (30, 60 and 120 minutes). After 60 mins reaction, no significant differences were found in the areas obtained for treatments (ANOVA test, p = 0.51; ɑ 0 5%). Then, the most favorable conditions for a laboratory workflow have been selected, leaving the reaction at room temperature (22°C +/- 3°C) for 60 minutes (Fig. S2). These conditions are of paramount importance for a high throughput routine method for GLY determination. As clearly concluded from Table 2, most reports overestimate the derivatization duration.

The stability of GLY-FMOC and AMPA-FMOC derivatives has been reported for 10 days [11]. However, we evidenced soft losses in peak height and area when analyzing the vials day-to-day. After 7 days of reinjection of calibration curve vials stored at room temperature in the dark, we found a decrease in GLY-FMOC area of 5.0% while for AMPA-FMOC it was 10%.

Based on these results, we recommend making fresh batch sequences that include blanks, calibration standards, quality controls and testing samples and analyzing them in the same analytical sequence.

Other minor changes to ISO 21458:2008 includes the use of DCM instead of diethyl ether in the cleanup step [7]. DCM is denser than an aqueous solution. Therefore, it enables a rapid separation of layers and direct sampling of aliquots from the upper phase.

We performed the validation using external calibration. This can be successfully performed with proper representative blank samples. Instead, ISO 21458:2008 suggests a standard addition method. In preliminary experiments, we studied LC-FLD selectivity and specificity in natural freshwater and ultrapure water samples. LC-FLD linearity was tested for GLY in the range 0.25 to 100 µg L^−1^ and between 1 to 100 µg L^−1^ for AMPA. Calibration curves of GLY-FMOC and AMPA-FMOC are obtained by spiking GLY and AMPA at different levels. These calibration standards are subjected to the complete analytical procedure, so they are finally corrected by the recovery. For LC-FLD, the lowest calibration level (LCL) with a signal-to-noise ratio (S/N) > 10 was selected as the limit of quantification (LOQ). LOQ was 0.25 µg L^−1^ for GLY and 1 µg L^−1^ for AMPA. The matrix effect (ME (%)) in freshwater for both compounds was 4.0%. Instrumental repeatability was analyzed in triplicate at the 10 µg L^-1^ levels, obtaining a relative standard deviation (RSD, %) at the Rt (min) of 0.16% for both compounds. Intra-day repeatability was 4.0% for both compounds for one analyst. In the case of inter-day repeatability, the procedure was performed by two different analysts after one week. In this case, the RSD for GLY and AMPA were 5.0%. Table 3 shows the validation results obtained for water analysis by LC-FLD.

**Table 3:** Figure of merits in freshwater by LC-FLD

	Freshwater			
	LOQ (µg L^−1^)	Dynamic range (µg L^−1^)	Matrix effect (%)	Repeatability RSD (%)
GLY	0.25	0.25 - 100	4	4
AMPA	1	1- 100	4	4

Here, we tested LC-FLD and LC-MS/MS in both freshwater and soil. LC-FLD identification is based on Rt (min) only. On the other hand, LC-MS/MS enables the identification of GLY and AMPA based on Rt (min) of highly specific ions of GLY-FMOC and AMPA-FMOC and their relative ion abundance. For freshwater analysis, detection by FLD was found to have higher sensitivity than MS/MS, reaching also lower LOQ values in the case of GLY.

For soil matrix, LC-FLD enabled GLY-FMOC analysis, but irresoluble chromatographic peaks overlap AMPA-FMOC peak. Different additional cleanup steps and chromatographic columns were tested without promising results. Considering the lack of specificity of AMPA-FMOC for soil analysis by LC-FLD, validation of GLY and AMPA residues in soil was performed by LC-MS/MS solely.

In the case of soil, linearity was tested for both analytes in the range 50 µg kg^−1^ to 1000 µg kg^−1^ for LC-MS/MS. Similarly, to freshwater samples, the calibration curves are corrected by analytical recovery. Here, LCL was selected as LOQ at 50 µg kg^−1^. The intra-day repeatability was evaluated for one analyst (n=4) being 4.0% for both compounds. The inter-day repeatability was performed by two different analysts with one week of difference obtaining an RSD of 12.0% for GLY and 13.0% for AMPA. Table 4 shows the validation results obtained for soil analysis by LC-MS/MS. Strong ion suppression is found in the soil matrix for both derivatives. ME (%) was estimated at -90% and -88% for GLY-FMOC and AMPA-FMOC, respectively. Based on these results we recommend the use of matrix matched calibration for soil analysis. One of the main difficulties is the use of GLY-FMOC and AMPA-FMOC standards. Only analytical grade GLY-FMOC standard is currently commercially available, which was additionally found to be unstable in aqueous solution. The sensitivity obtained with the reference standard was lower than that achieved by fresh derivatization of GLY.

**Table 4:** Figure of merits in freshwater and soil by LC-MS/MS

	Freshwater				Soil			
	LOQ (µg L^−1^)	Dynamic range (µg L^−1^)	Matrix effect (%)	Repeatability RSD (%)	LOQ (µg Kg^−1^)	Dynamic range (µg Kg^−1^)	Matrix effect (%)	Repeatability RSD (%)
GLY	1	1-100	0.3	5	50	50 - 1000	-90	12
AMPA	1	1-100	-1.6	3	50	50 - 1000	-88	12

Our approach relies on the use of external calibration of GLY and AMPA via a calibration function of FMOC derivatives prepared with blanks and spiked samples. On the other hand, other reports for MS/MS use isotopically labeled internal standard (ILIS) (see Table 2) [3,4,6,10,12].

This study highlights the complementarity of FLD and MS/MS for freshwater analysis in terms of qualitative and quantitative capabilities. However, the lack of selectivity of FLD for AMPA analysis in soil represents the main drawback. FLD offers a sensitive, robust and cost-affordable approach for laboratories looking for GLY analysis under a routine environment.

Of potential interest, can be the serial detection by FLD and MS/MS in one single instrumental setup. Online serial detection will support FLD determination and rapid GLY confirmation via selective MS/MS ions. FLD presents additional benefits in GLY quantitation in freshwater offering lower LOQs which might be relevant for environmental research studies. However, both online and offline workflows should be supported by routine MS/MS based confirmation of residues. A comparison of reported LOQs for different reported techniques using FMOC derivatization is presented in Table 2. As seen, competitive and fit-for-the-purpose LOQs are obtained from this study under both FLD and MS/MS conditions.

### Application to real samples

This analytical methodology was applied to soil and irrigation water samples from an experimental rice field with the objective of understanding the dynamics of glyphosate and its metabolite AMPA during the productive cycle (See exemplary Fig. S4). In this experimental field, GLY is applied before seeding. This method was employed to quantify GLY and AMPA in 153 freshwater samples and 75 soil samples, obtaining results in water ranging from LOQ to 14.6 µg L^−1^ for GLY and 36.6 µg L^−1^ for AMPA. In the case of agricultural soil samples, residues ranged from LOQ to 825 µg kg^−1^ for GLY and 238 to 1182 µg kg^−1^ for AMPA. From these results, it was possible to study and characterize the decay of GLY under experimental field conditions.

## Declaration of interests

The authors declare that they have no known competing financial interests or personal relationships that could have appeared to influence the work reported in this paper

## Appendix Supplementary materials

Image, application 1
