# Supplementary material for: Determination of glyphosate and AMPA in freshwater and soil from agroecosystems by 9-fluorenylmethoxycarbonyl chloride derivatization and liquid chromatography - fluorescence detection and tandem mass spectrometry
Source: MethodsX. 2022 May 13;9:101730. doi: 10.1016/j.mex.2022.101730 (PMC9157550; doi:10.1016/j.mex.2022.101730)
Supplement: Supplementary file 1 [file mmc1.docx]

**Supplementary material *and/or* Additional information:**

| Analyte | Rt (min) | Precursor ion (m/z) | Product ion (m/z) | DP (V) | CE (V) |
| --- | --- | --- | --- | --- | --- |
| GLY-FMOC | 6.5 | 390 | 168 | -35 | -17 |
|  | 6.5 | 390 | 150 | -35 | -36 |
| AMPA-FMOC | 10.5 | 332 | 110 | -30 | -11 |
|  | 10.5 | 332 | 136 | -30 | -23 |

Table S1: LC-MS/MS conditions for both compounds. Rt: retention time; DP: declustering potential; CE: collision energy.

|  | GLY-FMOC | | | | AMPA-FMOC | | | |
| --- | --- | --- | --- | --- | --- | --- | --- | --- |
| Detector | Slope | Intercept | R^2^ | LOQ (µg/L) | Slope | Intercept | R^2^ | LOQ  (µg/L) |
| FLD | 11235 | -890 | 0.99 | 0.25 | 43881 | -12958 | 0.99 | 1 |
| MS/MS | 1459 | 54 | 0.99 | 1 | 1698 | -386 | 0.99 | 1 |

Table S2: Comparison of calibration curves in water obtained by FLD and MS/MS


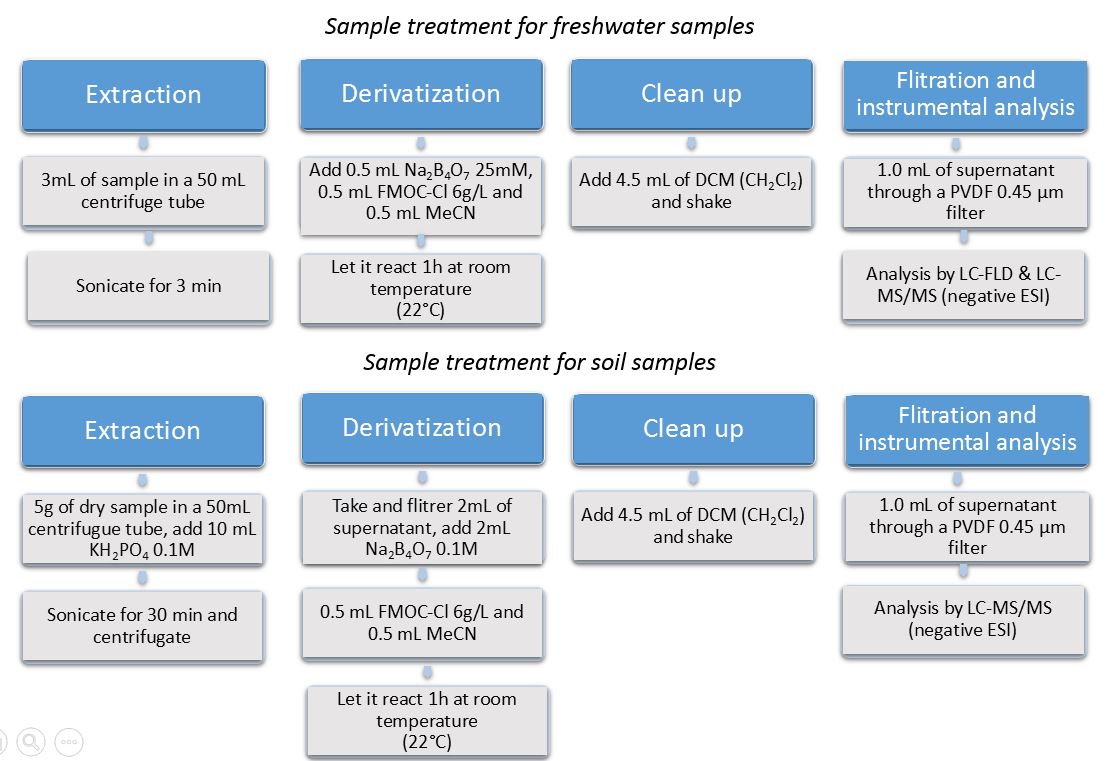


Figure S1: Analytical workflow of the customized methods for freshwater and soil.

**

**

Figure S2: LC-FLD peak of GLY showing a soft variation of area and retention time after 8 hours reinjection





Figure S3: Time vs temperature of derivatization experiment





Figure S4a: Results obtanied in soil real samples with the proposed method





Figure S4b: Results obtanied in freshwater real samples with the proposed method.
